# Appendicitis with appendicular atresia: a rare presentation

**DOI:** 10.11604/pamj.2015.20.57.4500

**Published:** 2015-01-22

**Authors:** Irfan Masood, Zain Majid, Ali Rafiq, Saba Fatima, Osama Bin Zia Siddiqui

**Affiliations:** 1 Department of Surgery, Civil Hospital, Karachi, Pakistan

**Keywords:** Appendicitis, appendicular atresia, appendectomy

## Abstract

Acute appendicitis is the most common acute surgical condition; making appendectomy the most commonly performed emergency surgical procedure in the world. Anomalies of the appendix are relatively uncommon. However, their presence may alter the course of pre-operative diagnosis and the surgical treatment provided, leading to medico-legal issues in certain cases as well. We hereby present the case of a 17 year-old female who had the suggestive signs, symptoms and investigations of appendicular lump. She was managed according to the Ochsner-Sherren regimen and then underwent interval open appendectomy 6 weeks later. During the procedure, the findings of a 5 cm long appendix were noted. The base of the appendix was attached to the caecum, however there was complete mucosal discontinuity between the base and the remaining portion of the appendix. A fibrous strand connected the two blind ending parts together. After thorough literature search, the authors concluded that this is only the fourth reported case of appendicular atresia ever to have been reported. Considering the rarity of this finding we feel this could be of valuable interest to surgeons and readers alike

## Introduction

Acute appendicitis is the most common acute surgical condition; making appendectomy the most frequently performed surgery in emergency setting [1–3]. Delayed presentation may be followed by the development of appendicular lump in 2-10% of cases [4]. This mass is composed of inflamed appendix, edematous cecal wall, edematous loop of ileum, and the omentum [5]. The management of appendicular lump usually consists of conservative treatment followed by delayed appendectomy [6]. The appendix, considered a vestigial organ, is retrocecal or retrocolic in most of the cases [7]. Other documented positions include the pelvic, subcecal, pre-ileal, splenic and ectopic positions, enlisted here in decreasing order of frequency [7]. Appendiceal anomalies are uncommon, however, they may interfere with preoperative diagnosis and surgical treatment, and may have medico-legal consequences.

## Patient and observation

A 17-year old female presented to the surgical unit through the emergency department (ED) with active complaints of pain in the right iliac fossa (RIF) for 3 days. The pain started in the peri-umbilical region, shifting later to the right iliac fossa (RIF). It was acute in onset, moderate to severe in intensity and continuous in nature. It was associated with anorexia, nausea and two episodes of non-bilious vomiting. The patient was also complaining of low-grade fever (100.6°F) with no rigors or chills. On examination, the patient was oriented to time, place and person and had a normal blood pressure but was tachycardic (104 beats per minute). Abdomen was soft, with tenderness and rebound tenderness in RIF and a 3x3 cm lump was felt in the RIF. Rest of abdominal and systemic examination were normal. Hematologic investigations showed haemoglobin of 13.8 g/dL, total leukocyte count (TLC) of 10,800 x10^9^/L and a normal platelet count. Serum electrolytes and urinalysis were within normal limits and pregnancy test was negative. Ultrasound abdomen (US abdomen) showed findings consistent with that of an appendicular lump. She was conservatively managed by Ochsner-Sherren regimen after which her symptoms resolved in 4 days. Upon discharge she was advised for interval appendicectomy 6 weeks later.

**Figure 1:**
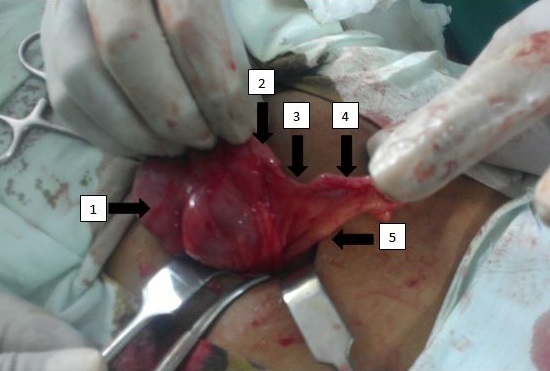
Appendicular atresia (arrow 1= cecum, arrow 2= base of appendix, arrow 3= fibrous strand ie the atretic portion connecting the base with the remaining of appendix, arrow 4= remaining of appendix including the tip, arrow 5= mesoappendix)

The patient underwent open appendicectomy with intra-operative findings of an approximately 5-cm long appendix. The base of appendix was attached to the cecum but there was complete mucosal discontinuity between the base and the rest of appendix (Figure 1). The separated blind-ended parts were connected to each other by a single fibrous strand. Mesoappendix containing appendicular artery was visible anchoring the appendix to cecum. Mesoappendix was tied and divided and appendix was removed. Macroscopically, no fecolith or mass were identified on either ends. Rest of bowel, mesentery, and pelvic organs were normal. The sample was sent for histopathologic examination, which showed chronic appendicitis with no granuloma, carcinoid, or malignancy. The patient was discharged with no immediate or late post-operative complications.

## Discussion

The diagnosis of acute appendicitis is generally made on the patient's history, clinical examination, and supporting laboratory and radiological findings. Developmental anomalies of the vermiform appendix can hence complicate the clinical and surgical presentations of patients presenting with acute abdomen [8].

Developmental anomalies of the appendix are extremely rare. Duplication of appendix has an incidence of 0.004% [9, 10], while the incidence of appendicular diverticulosis found in appendectomy specimens ranges from 0.004% to 2.1% [11]. Congenital absence of appendix i.e. appendicular agenesis has a reported incidence of 1 in 100,000 cases [12–14]. Others include horse-shoe appendix [15], malrotation [16], and a single reported case of triplication of appendix. As per our knowledge, the case that we have hereby reported is only the fourth case of appendicular atresia.

Woywodt A et al. in 1998, reported the first case of appendicular atresia, which was associated with multiple jejunal atresia in a 4-year old boy with intestinal obstruction. Nichat et al., in 2009, reported the second case of appendicular atresia, in which the term “double-blind” appendix was used. Like our case, the patient was initially managed conservatively and then underwent interval appendectomy. However, the sequence of mucosal discontinuity of appendix was different from our case. In the case reported by Nichat et al. the sequence was cecum-fibrous strand (i.e. the atretic portion)-appendix, while in our case the sequence was cecum- base of appendix- fibrous strand-remaining of appendix. Yaylak F et al. in 2013, reported another case of appendicular atresia in a 59-year old male with acute appendicitis. The appendix was approximately 10 cm proximal to ileocecal valve. The tip of appendix was inflamed, while the lumen had ended blindly without a connection to the cecum, but supplied by ileocecal artery. Hence, our case is only the fourth case of appendicular atresia and we believe that it is a valuable addition to this rare pool of developmental anomalies.

## Conclusion

Hence, our case is only the fourth case of appendicular atresia and we believe that it is a valuable addition to this rare pool of developmental anomalies.
